# Glutamate Transporter GLT-1 Upregulation Attenuates Visceral Nociception and Hyperalgesia via Spinal Mechanisms Not Related to Anti-Inflammatory or Probiotic Effects

**DOI:** 10.1155/2011/507029

**Published:** 2011-12-12

**Authors:** Y. Lin, K. Roman, K. D. Foust, B. K. Kaspar, M. T. Bailey, R. L. Stephens

**Affiliations:** ^1^Department of Physiology and Cell Biology, The Ohio State University, Columbus, OH 43210, USA; ^2^Department of Neuroscience, The Ohio State University, Columbus, OH 43210, USA; ^3^The Research Institute at Nationwide Children's Hospital, Columbus, OH 43210, USA; ^4^Department of Oral Biology, The Ohio State University, Columbus, OH 43210, USA

## Abstract

Visceral pain is the most common reason for physician visits in US. Glutamate is the major excitatory neurotransmitter and mediates visceral nociceptive neuro-transmission and hypersensitivity. Removal of extracellular glutamate is predominantly mediated by glial glutamate transporter-1 (GLT-1). The pharmacological approach to up-regulate GLT-1 by 1 week administration of ceftriaxone (CTX) has been successful to mitigate visceral nociception. The present study shows that intrathecal delivery of selective GLT-1 antagonist dihydrokainate reversed CTX-blunted visceral nociceptive response, suggesting a spinal site of action. The role of GLT-1 up-regulation in animal models of colitis was studied. CTX treatment reversed TNBS-induced visceral hypersensitivity. In addition, CTX treatment initiated one week after the onset of DSS-induced visceral inflammation also attenuated visceral hypersensitivity, revealing a potential therapeutic effect. Cephalothin, a cephalosporin antibiotic lacking GLT-1 induction activity, failed to attenuate visceral nociception. CTX-induced changes in fecal microbiota do not support a role of probiotic effects in mitigating visceral nociception/hypersensitivity. Finally, adeno-associated virus serotype 9-mediated GLT-1 over-expression was effective to mitigate visceromotor response to 60 mmHg colo-rectal distension. These studies indicate that GLT-1 over-expression is a novel and effective method to attenuate visceral nociception, and is deserving of further study as a translationally relevant approach to treat visceral pain.

## 1. Introduction

Visceral pain, defined as pain associated with the internal organs, is a major clinical problem affecting up to 25% of the general US population and is the most common reason for physician visits in the USA. Lower pain thresholds to aversive stimuli are common, due to visceral organ primary afferent sensitization, hyperexcitability of second-order pain-transmitting neurons, or dysregulation of descending modulation of nociception [1]. These pain categories are the most refractory to effective treatment, in part due to its diverse nature and the contribution of distinct and redundant mechanisms that are incompletely understood and differ from the better studied somatic nociceptive system [2]. Inflammation plays a significant role in the peripheral and central sensitization seen in a subgroup of subjects [3].

A series of exciting recent findings in the subclinical study of visceral pain shows that a novel strategy to decrease synaptic glutamate by upregulating the physiologically dominant glutamate transporter GLT-1 is effective to mitigate visceral nociception [4–6]. Evidence suggests that the expression of new protein is stimulated by the *β*-lactam antibiotic ceftriaxone (CTX) [7, 8]. Regarding clinical potential of this approach, pharmacologic GLT-1 overexpression utilizing the cephalosporin antibiotic ceftriaxone has reached Phase III clinical trials for the treatment of amyotrophic lateral sclerosis [9], http://clinicaltrials.gov/ct2/show/NCT00349622?term=Ceftriaxone&rank=4, and is being explored for other CNS disorders [10]. In this study of antinociceptive efficacy, experiments are designed to determine (1) the site of action of CTX-mediated GLT-1 upregulation, (2) whether viral transfection of GLT-1 utilizing translationally superior adenoassociated virus serotype 9 (AAV9) mitigates visceral nociception, (3) the effectiveness of GLT-1 upregulation to mitigate inflammogen-mediated visceral hyperalgesia, and (4) the relative role of enhanced glutamate transporter activity, anti-inflammatory action, and changes in intestinal microbiota to mitigate visceral nociception. The results (i) confirm a spinal site of action of CTX-induced enhanced glutamate transporter activity to mitigate visceral nociception due to colonic distension, (ii) reveal effectiveness of AAV9-mediated transfection of GLT-1 to blunt visceral nociception and (iii) demonstrate effectiveness of both preemptive and therapeutic GLT-1 overexpression to mitigate inflammogen-mediated visceral hyperalgesia while not affecting murine locomotor activity.

## 2. Methods

### 2.1. Animals

Two-to-three-month old FVB/N mice of both genders were used for the behavioral studies. CR57/BL mice were used for the adenoassociated virus (AAV9) studies. Mice were maintained on a twelve-hour day–twelve-hour night cycle and were housed in groups of five with water and food. All protocols were approved by the Institutional Animal Care and Use Committee in the Ohio State University and adhered to the guidelines of the Committee for Research and Ethical Issues of the International Association for the Study of Pain.

### 2.2. Drug Administration

Ceftriaxone (CTX; Sigma, St. Louis, Mo, USA) was prepared in saline and was administrated intraperitoneally 200 mg (302 *μ*moles)/Kg/day in a 10 ml/kg volume for one week. Cephalothin (CLT) was administered in an equimolar dose, and in the same route and duration as CTX. Normal saline was used as control injections for these experiments. 

### 2.3. AAV9-GLT-1 Transfection

The human GLT-1 cDNA was subcloned from a construct obtained as a gift from Dr. Jeffrey Rothstein (Johns Hopkins University). The Kaspar Laboratory routinely achieves >1013 viral particles from AAV preparations [11]. For neonate injections in 19–26-d-old pups, a light microscope was used to visualize the tail vein. Vector solution was drawn into a 3/10 cc 30-gauge insulin syringe. The needle was inserted into the vein, and the plunger was manually depressed. Virus injections were in a total volume of 100 ml of PBS supplemented with 0.001% pluronic F68. A total of 4 × 10^11^ DNase-resistant particles of scAAV9-CB-GLT-1 were injected. A correct injection was verified by noting blanching of the vein. After the injection, pups were returned to their cage. Experiments were performed in 8-week-old adult mice.

### 2.4. Colorectal Distention

Among the pseudoaffective responses commonly employed in rodents as an index for nociception is the visceromotor response to colorectal distension (CRD). CRD was performed as described previously [12]. Briefly, animals were surgically implanted with two electromyography (EMG) electrodes fashioned from insulated silver wire in the external oblique muscle. Three days after recovery from surgery, the visceromotor response to colorectal distention was elicited by intracolonic balloon distention in triplicate at 15, 30, 45, and 60 mmHg pressure. The data was analyzed as the number of electromyographic spikes above baseline using Spike2 software (CED, UK). 

### 2.5. Intrathecal Administration

In some experiments, one hour before colorectal distention, animals were lightly anesthetized by isoflurane. The hair in the midline of the back was shaven, and the locus of the cauda equina was determined as described previously [13]. A 30-gauge needle connected to a 10 *μ*l Hamilton syringe was inserted through the L5-L6 vertebra to reach the dura at the lumbosacral spinal level. A characteristic tail flick occurred after correct placement of the needle. 5 *μ*l of vehicle or dihydrokainate (DHK) at different concentrations (3, 0.3, 0.003 mM) was delivered into the intrathecal space over one minute, and the animals were prepared for colorectal distension. 

### 2.6. Intracisternal Administration

In some experiments, one hour before colorectal distention, animals were lightly anesthetized by isoflurane. Hair was shaven on the dorsal aspect of the neck and was removed, and a 0.5 cm diameter steel bar was put underneath the neck. A 30-gauge needle connected to a 50 *μ*l Hamilton syringe was carefully advanced to penetrate the allantoin-occipital membrane to access the cisterna magna. Vehicle, 0.3 or 3.0 mM dihydrokainate (DHK), was then delivered in a 5 *μ*l volume into the cistern magna, and the animals were prepared for colorectal distension. 

### 2.7. Colonic Inflammation with Trinitrobenzene Sulfonic Acid (TNBS)

Two groups of animals were treated with intracolonic TNBS (7 mg/ml) and another two cohorts treated with intracolonic vehicle prepared in 50% ethanol and delivered through a syringe attached to a lubricated polyethylene catheter (PE20) two centimeters proximal to the anus in a 100 *μ*l volume. After instillation (day 0), mice were held head-down by lifting up the tail for 1 min to ensure exposure of distal colon to injectate. CTX 200 mg/Kg/day or vehicle injections were administered intraperitoneally at 10 : 00 am days 0–6. Mice were utilized on day 7 for behavioral tests, myeloperoxidase (MPO) assay, and immunoblot analysis.

### 2.8. Colonic Inflammation with DSS

Two groups of animals were administered five mililliters daily of normal drinking water and another two groups administered 4% dextran sodium sulfate (DSS) dissolved in the drinking water daily for 1 week (days 0 to 6). Mice receiving 4% DSS typically develop colitis by day 7 [14]. All animals were then switched to normal drinking water for the remainder of the study. Ceftriaxone (200 mg/Kg/day; ip) was then administered therapeutically for one week (days 7–13) in one cohort treated with 4% DSS and one cohort treated with normal drinking water. Similarly, vehicle was injected on days 7–13 in one cohort treated with 4% DSS and one cohort treated with normal drinking water. Mice were utilized on day 14 for behavioral and biochemical analysis.

### 2.9. Myeloperoxidase (MPO) Assay

As a measure of colonic inflammation, 1 cm of the distal colon was dissected from animals and samples were minced and homogenized 30 strokes by a motor-driven grinder in 1 ml ice-cold 50 mM potassium phosphate buffer (pH 6.0) with 0.5% hexadecyltrimethylammonium bromide (Sigma). Samples subsequently underwent 30 s sonication and three freeze-thaw cycles in liquid nitrogen. After brief sonication, samples were centrifuged at 10,000 rpm for 10 min at 4°C. Supernatant was collected to measure MPO activity. 200 *μ*l of 50 mM potassium phosphate (pH 6.0) containing 0.167 mg/ml O-dianisidine dihydrochloride (Sigma) and 0.0005% hydrogen peroxide was loaded as reaction buffer in a 96-well plate. 20 *μ*l of supernatant sample was then mixed with reaction buffer. Two minutes later, absorbance was read at 460 nm by a spectrometer (PowerWave XS, BioTek). Protein concentration was accessed by adding the 1 *μ*l sample into 200 *μ*l coomassie blue buffer (Pierce) in the same 96-well plate, and absorbance was read at 595 nm. The unit of MPO was defined by the change in 460 nm absorbance per mg of protein sample in two minutes.

### 2.10. Immunoblotting

Briefly, 20 *μ*g of protein sample dissected from mice lumbosacral spinal cord was sonicated, loaded in 8% SDS-PAGE gel, and transferred to nitrocellular membranes. After one-hour blocking, the membranes underwent over-night primary antibody (rabbit anti-GLT-1 antibody, 1 : 1000; rabbit anti-actin antibody, 1 : 1000, Santa Cruz) and one-hour secondary antibody (HRP-conjugated rabbit IgG, 1 : 3000, Santa Cruz) incubation, followed by X-film exposure with Enhanced Chemiluminescent Substrate (Pierce).

### 2.11. Rotarod Test

Mice were trained on an accelerating rotarod (Columbus Instrument, Columbus, OH) 3 times per day for 3 days. The cylinder of the rotarod was 71 cm long, and its diameter was 3.2 cm. Mice were allowed to explore the rotarod for 2 min without rotation. The velocity of the rod was set to increase from 0 to 40 rpm in 5 min. Mice with stable 300 s performance on the 3rd day were used later. At the end of baseline testing, animals were divided into two groups and one group treated with CTX 200 mg/Kg/day and the other cohort administered vehicle i.p. After this one-week treatment, mice were tested three times per day for 3 days with 1-hour interval, following the same protocol. The latency to fall off the apparatus in the three trials was recorded, and the 3-day data from individual mouse was averaged. Significant difference was assessed by using Student's *t*-test. 

### 2.12. Statistics

Multiple group differences were analyzed by ANOVA followed by post-hoc LSD analysis.

## 3. Results

### 3.1. Intrathecal (it) DHK Treatment Dose-Dependently Reversed Attenuated Visceral Nociceptive Response Produced by GLT-1 Overexpression

**Figure 1:**
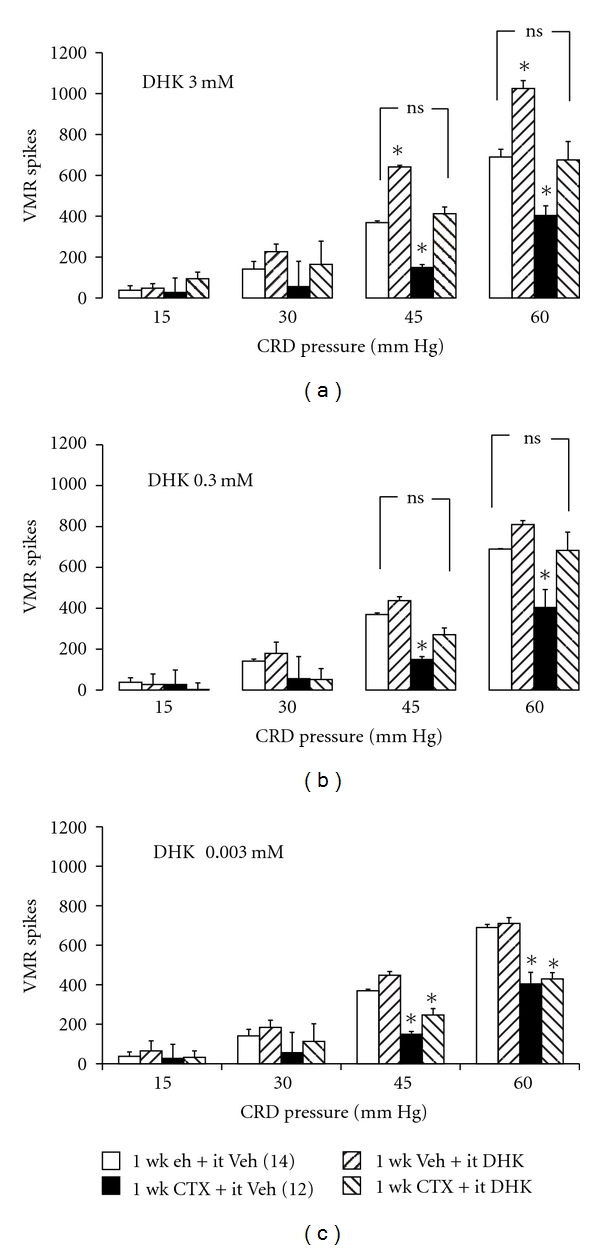
Comparison of visceromotor responses (VMR spikes) to colorectal distension (CRD) between (i) animals treated 1 week with vehicle (intraperitoneal; ip) and vehicle (intrathecal; it) given 1 hour before CRD, (ii) animals treated 1 week with vehicle (ip) and dihydrokainate (DHK 3 mM, it; *n* = 4 (a), DHK 0.3 mM, it; *n* = 8 (b), and DHK 0.003 mM, it; *n* = 7 (c)) given 1 hour before CRD, (iii) animals treated 1 week with ceftriaxone (CTX 200 mg/kg, ip) and vehicle (it) given 1 hour before CRD, and (iv) animals treated 1 week with (CTX 200 mg/kg, ip) and (DHK 3 mM, it; *n* = 6 (a), DHK 0.3 mM, it; *n* = 8 (b), and DHK 0.003 mM, it; *n* = 7 (c)) given 1 hour before CRD. Data from the 1-week vehicle + it vehicle group and 1-week CTX + it vehicle group are from the same animals in (a)–(c). *P < 0.05 compared to the 1-week Veh + it Veh group. Intrathecal DHK reversed the attenuated visceromotor response caused by 1-week CTX treatment at 3 and 0.3 mM but not 0.003 mM doses.

 GLT-1 overexpression induced by 1-week CTX treatment produced a significant 41–60% reduction in the visceromotor response to 45 and 60 mmHg colorectal distention (1-week CTX + it vehicle), compared to animals treated with 1-week vehicle + it vehicle (Figures 1(a)–1(c)). To assess whether spinal GLT-1 overexpression mediates attenuation of the visceromotor response to colorectal distension, the selective GLT-1 antagonist dihydrokainic acid (DHK; 5 *μ*l, 3 mM) was injected into the intrathecal space (it) one hour before the graded visceromotor response to colorectal distension was elicited. In animals with GLT-1 overexpression produced by 1-week CTX, intrathecal DHK (1-week CTX + it DHK 3 mM) reversed the blunted visceromotor response produced by GLT-1 overexpression (1-week CTX + it vehicle; Figure 1(a)). 

Notably, the 1-week vehicle + it DHK 3 mM group (Figure 1(a)) produced an augmented visceromotor response to 45 and 60 mmHg colorectal distension compared to controls (1-week vehicle + it vehicle), likely due to enhanced extracellular glutamate [15]. To further investigate the dose-response relationship of intrathecal DHK to reverse blunted visceromotor response caused by GLT-1 overexpression, a tenfold lower dose of DHK (0.3 mM) was injected one hour prior to colorectal distention (Figure 1(b)). The group treated with 1-week vehicle + the lower dose (0.3 mM) of it DHK was still able to reverse the blunted visceromotor response to 45 and 60 mmHg colorectal distension seen in the 1-week CTX + it vehicle group (Figure 1(b)), even though the 0.3 mM dose of it DHK did not produce enhanced visceromotor response to 45 and 60 mmHg colorectal distension compared to the 1-week vehicle + it vehicle cohort (Figure 1(b)). Moreover, the DHK-mediated reversal of the inhibitory effect of 1-week CTX was lost after a further 100-fold decrease in the dose of DHK (1-week CTX + it DHK 0.003 mM) (Figure 1(c)). Thus, the data suggests a DHK-mediated dose-dependent reversal of the blunted visceromotor response to colorectal distension caused by GLT-1 overexpression.

### 3.2. Intracisternal (ic) DHK Pretreatment Did Not Reverse Attenuated Visceral Nociceptive Response Caused by GLT-1 Overexpression

**Figure 2:**
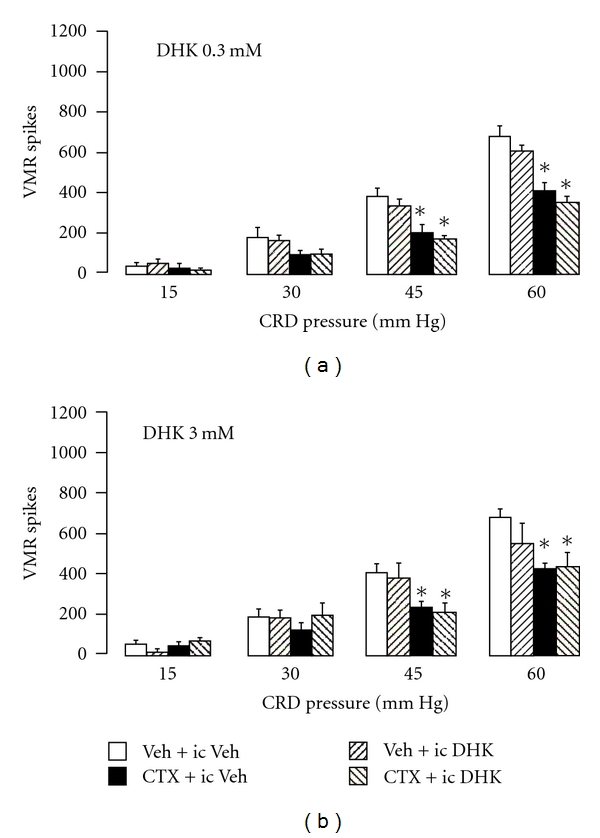
Comparison of visceromotor responses (VMR spikes) to colorectal distension (CRD) between (i) animals treated 1 week with vehicle (intraperitoneal; ip) and vehicle (intracisternal; ic) given 1 hour before CRD; *n* = 7, (ii) animals treated 1 week with vehicle (ip) and dihydrokainate (DHK 0.3 mM, ic; *n* = 6 (a) and DHK 3 mM, ic; *n* = 6 (b), given 1 hour before CRD; *n* = 7, (iii) animals treated 1 week with ceftriaxone (CTX 200 mg/kg, ip) and vehicle (ic) given 1 hour before CRD, and (iv) animals treated 1 week with (CTX 200 mg/kg, ip) and (DHK 0.3 mM, ic; *n* = 6 (a) and DHK 3 mM, ic; *n* = 4 (b), given 1 hour before CRD. Data from the 1-week vehicle/ic vehicle group and 1-week CTX/ic vehicle group are from the same animals in (a) and (b). *P < 0.05 compared to the 1-week Veh + ic Veh group. Intracisternal DHK was ineffective to reverse the attenuated visceromotor response caused by 1-week CTX treatment.

 Given the putative role of hindbrain glutamate receptors to modulate pain transmission [16], the role of enhanced activity of supraspinal GLT-1 in mediating inhibition of the visceromotor response was investigated. DHK was delivered into the cisterna magna one hour prior to colorectal distension (Figures 2(a) and 2(b)) at doses 3.0 and 0.3 mM previously shown effective to reverse the inhibitory effect of GLT-1 overexpression after intrathecal administration (Figures 1(a) and 1(b)). Neither dose of intracisternal DHK reversed the attenuated visceromotor response in 1 week CTX + it vehicle-treated animals (Figures 2(a) and 2(b)). These data suggests that GLT-1 overexpression in the hindbrain does not mediate the attenuation of the visceromotor response to colorectal distension.

### 3.3. GLT-1 Overexpression Reversed TNBS-Induced Visceral Hyperalgesia

**Figure 3:**
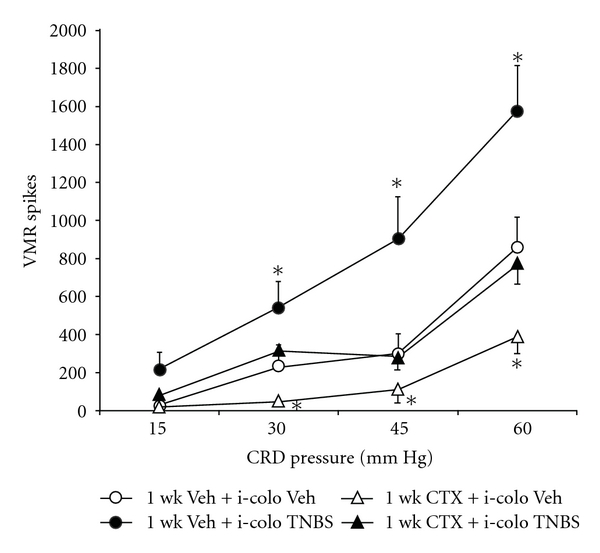
The effect of GLT-1 overexpression on the intracolonic (i-colo) TNBS-sensitized visceromotor response to graded colorectal distension were compared in female mice. In animals treated with 1-week vehicle and then intracolonic TNBS (100 *μ*l, 7 mg/ml) (●), administered 1 week before the study, significant increased visceromotor response to colorectal distension (*P < 0.05) was observed, compared to 1-week vehicle and intracolonic vehicle (○). One-week CTX treatment abolishes the enhanced visceromotor response to colorectal distension caused by intracolonic TNBS (▲). *n* = 7-8/group. *P < 0.01 compared to 1-week vehicle + intracolonic vehicle group.

Colonic inflammation causes visceral hyperalgesia [17] and this association is clinically relevant. To test the effectiveness of GLT-1 overexpression on inflammogen-mediated visceral hyperalgesia, the inflammogen TNBS (0.1 ml; 7 mg/ml) was instilled into the distal colon 1 week before the visceromotor response to colorectal distension was elicited. TNBS was administered at day 0, and CTX (200 mg/Kg, ip) was administrated at 10 : 00 am daily from days 0 through 6. Mice were exposed to colorectal distension and/or tissues harvested at day 7. Intracolonic TNBS resulted in a significant increase (42–69%) in the visceromotor response to 30, 45 and 60 mmHg colorectal distension, compared to control animals (Figure 3). GLT-1 overexpression by 1-week CTX treatment reversed colitis-enhanced visceromotor response to the 30, 45 and 60 mmHg CRD, compared to TNBS-treated animals (Figure 3). 

Glutamate receptor activation mediates colonic inflammation [17, 18] as well as CNS events that mediate hyperalgesia [16]. Thus, the relative role of the anti-inflammatory effects of decreased glutamatergic tone by GLT-1 overexpression was explored. Myeloperoxidase (MPO) activity as an index of inflammation [19] was measured in distal colon samples 7 days after intracolonic TNBS treatment to assess effects of concomitant 1-week CTX on colonic inflammation. Intracolonic TNBS treatment led to a significant increase in MPO activity, compared to vehicle-treated animals (Table 1; *P* < 0.005). 1-week CTX treatment inhibited the enhanced MPO activity produced by colonic TNBS administration (Table 1; *P* < 0.005). These data suggest that pharmacologic GLT-1 overexpression was able to attenuate both the intracolonic TNBS-elevated visceromotor response to colorectal distension (Figure 3), as well as the induced inflammatory response. 

### 3.4. Effect of a Cephalosporin Antibiotic Cephalothin (CLT) Not Enhancing GLT-1 Expression on Visceral Hyperalgesia Induced by TNBS

**Figure 4:**
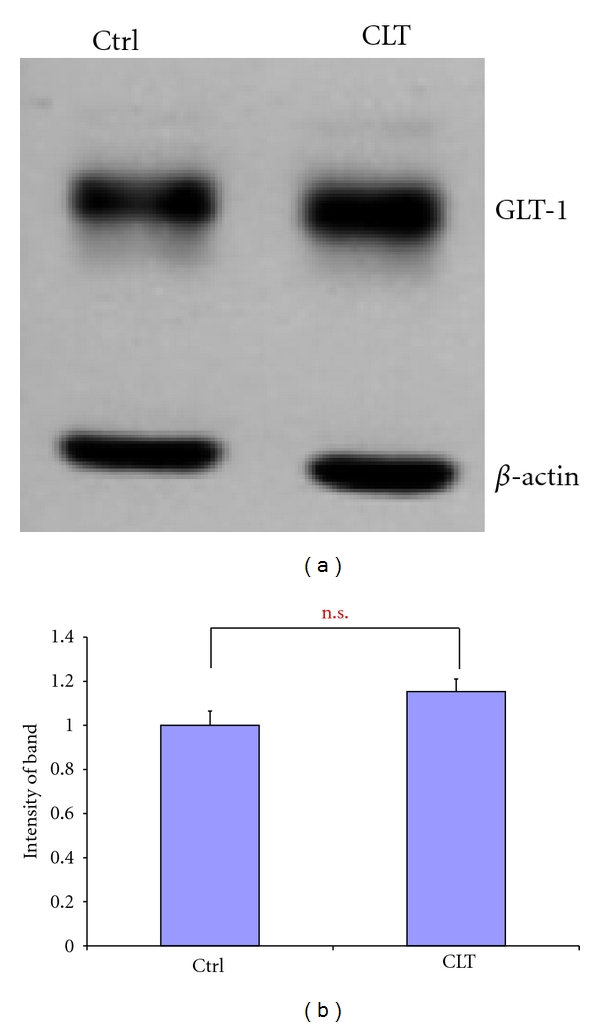
CLT treatment did not alter spinal GLT-1 expression. (a) Representative western blot of GLT-1 protein levels in the spinal cords of CLT- and vehicle-treated mice. (b) Intensity analysis of western bands showed that one-week CLT treatment (200 mg/Kg/day) did not alter GLT-1 protein expression compared to controls (*n* = 3, each group; *P* > 0.05).

**Figure 5:**
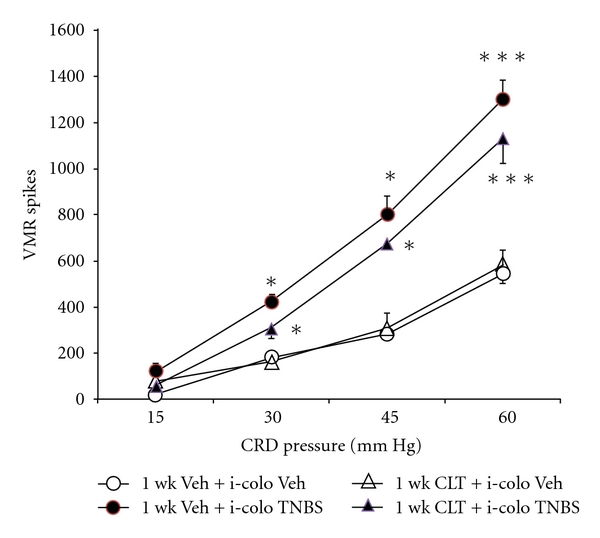
The effect of 1-week cephalothin (CLT) on the intracolonic (colo) TNBS-sensitized visceromotor response to graded colorectal distension was compared in female mice. In animals treated with 1-week vehicle and then intracolonic TNBS (100 *μ*l, 7 mg/ml) (●), administered 1 week before the study, significant increased visceromotor response to colorectal distension (*P < 0.05) was observed, compared to 1-week vehicle and intracolonic vehicle (○). One-week CLT treatment did not attenuate the enhanced visceromotor response to colorectal distension caused by intracolonic TNBS (▲). *n* = 6-7 per group. *P < 0.01 compared to 1-week vehicle + intracolonic vehicle group.

To further explore the putative role of the anti-inflammatory effect of cephalosporin antibiotics to mediate antinociception, cephalothin (CLT), a cephalosporin antibiotic not effective to enhance GLT-1 expression (Figure 4) was utilized to investigate its effect on the enhanced visceromotor response induced by intracolonic TNBS. Immunoblots show that, in contrast to 1-week CTX, administration of equimolar 1-week CLT ((302 *μ*mole) 126 mg/Kg/day) did not increase the GLT-1 protein level in the spinal cord, compared to vehicle-treated mice (Figure 4). In contrast to 1-week CTX, 1-week CLT treatment did not significantly attenuate intracolonic TNBS-induced hyperalgesia (Figure 5), but significantly reduced the enhanced MPO response caused by intracolonic TNBS (Table 2), These data taken together suggest that the anti-inflammatory property of cephalosporins alone do not inhibit the hyperalgesia produced by inflammogen TNBS and implicates the GLT-1 overexpression property of 1-week CTX as a mediator of the antinociceptive effect.

### 3.5. Therapeutic GLT-1 Overexpression Reversed Ongoing Visceral Hypersensitivity Induced by DSS

**Figure 6:**
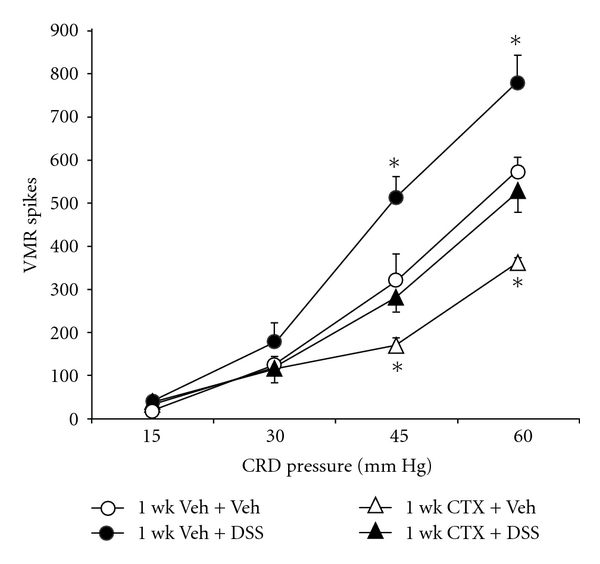
The effect of GLT-1 overexpression on the dextran-sodium-sulfate- (DSS-) sensitized visceromotor response to graded colorectal distension was compared in female mice. In animals treated with oral 4% DSS for 1 week followed by 1-week daily vehicle injections (ip) (●), significant increased (36–60%) visceromotor response to colorectal distension (*P < 0.05) was observed, compared to 1-week vehicle and intracolonic vehicle (○). One-week CTX treatment abolishes the enhanced visceromotor response to colorectal distension caused by oral 4% DSS for 1 week (▲). *n* = 7–9 per group. *P < 0.01 compared to 1-week vehicle + intracolonic vehicle group.

A key question related to potential therapeutic utility of antinociceptive strategies to overexpress GLT-1 is its effectiveness after the initiation of algesia. This study utilized the DSS-induced colitis model, where 5 ml of 4% DSS in the drinking water was provided daily to two groups of mice for one week. Comparison cohorts of two groups of mice were administered normal drinking water for week one. All animals were returned to normal drinking water for week 2, and GLT-1 overexpression was produced by 1-week CTX (200 mg/Kg, ip daily) from day 7 through day 13 in two groups of mice (1-week oral DSS and normal drinking water control), and two groups (1-week oral DSS and normal drinking water control) were treated with ip vehicle daily for 1 week. In response to colorectal distension performed at day 14, DSS treated animals displayed a significantly increased (36–60%) visceromotor response to 45 and 60 mmHg colorectal distension, compared to control animals (Figure 6). At day 14, DSS-treated animals also exhibited severe colitis in the distal colon, as measured by a significant increase in MPO activity compared to control animals (Table 3). Therapeutic CTX treatment significantly reversed the DSS-enhanced visceromotor response to the 45 and 60 mmHg CRD tests, compared to DSS-treated animals (Figure 6). However, 1-week CTX treatment after the establishment of colitis [20] by 4% DSS still produced significantly greater colonic MPO activity compared to 1-week-vehicle-treated controls (Table 3). These data implicate GLT-1 overexpression by 1-week CTX treatment as a mediator of attenuation of DSS-evoked hyperalgesia. 

### 3.6. GLT-1 Overexpression: Effects on Motor Function

An important question related to possible clinical utility of CTX treatment is to evaluate possible untoward effects caused by GLT-1 overexpression. Previous reports show a lack of effect on acute nociceptive and selective behavioral tests in rodents [5, 7]. To assess possible effects on general locomotion, the rotarod test was employed to evaluate whether GLT-1 upregulation by one-week CTX treatment alters movement coordination and balance. Animals were trained on the rotarod apparatus daily for 1 week; during this time one-half of the animals were injected with vehicle and the other half were administered CTX (200 mg/kg) daily. There was no significant difference in the falling latency between CTX and vehicle-treated animals (latency to fall (seconds): vehicle, 251 ± 11; CTX, 292 ± 5). These data suggested that GLT-1 upregulation by 1-week CTX treatment did not alter the motor behavioral function in mice. 

### 3.7. Ceftriaxone and Cephalothin: Comparison of Effects on Intestinal Flora

**Figure 7:**
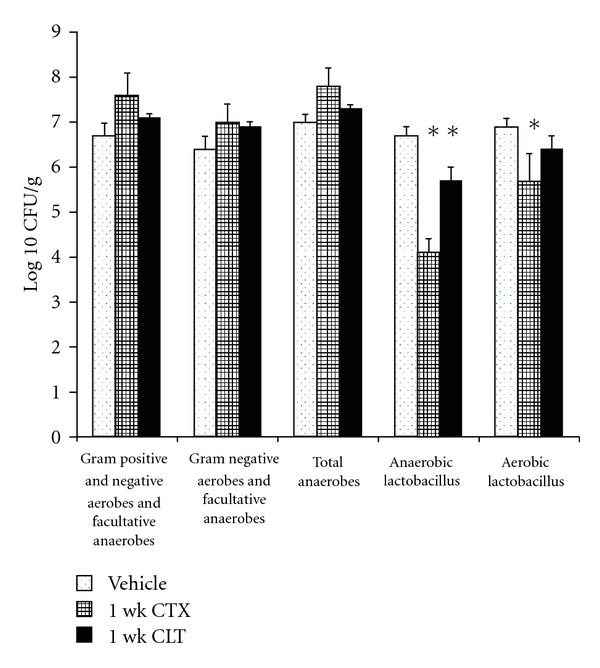
Comparison of the effect of 1-week vehicle, CTX or CLT on mouse fecal pellet biota. The data are the means + SEM of levels of flora types indicated on the *x*-axis. *n* = 8–10 per treatment group. *P < 0.05 compared to vehicle-treated cohorts.

Emerging evidence suggests that changes in intestinal microflora can mediate alterations in gastrointestinal function, including visceral hypersensitivity [21]. To assess a putative role of changes in intestinal flora to mediate antinociception caused by ceftriaxone (CTX) or cephalothin (CLT), fecal biota were quantitated after 1-week administration of either agent, and compared to controls. Figure 7 shows that both 1-week CTX and CLT cohorts exhibited a significant reduction (39% and 15%, resp.) in anaerobic lactobacillus, compared to the control group. In addition, the 1-week CTX but not 1-week CLT cohort exhibited a significant reduction (17%) in aerobic lactobacilli, compared to control group. Since reductions in lactobacilli are associated with enhanced visceral hypersensitivity [21] the CTX-induced reduction in intestinal lactobacilli appears not to explain its antinociceptive effects. The numbers of (1) total anaerobes, (2) Gram-positive/Gram-negative aerobes and facultative anaerobes, and (3) gram negative aerobes and facultative anaerobes were not different in groups treated with 1-week CTX or CLT compared to controls.

### 3.8. AAV9-Mediated GLT-1 Transfection Attenuated the Visceromotor Response to Colonic Distension

**Figure 8:**
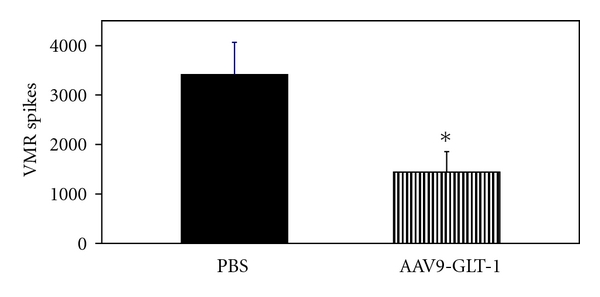
The visceromotor response to 60 mmHg colorectal distension in mice treated at day 21 with intravenous PBS or AAV9-GLT-1. The data show a 62% reduction in the nociceptive response after transfection with GLT-1. *n* = 10, each group; *P < 0.05 compared to control.

**Figure 9:**
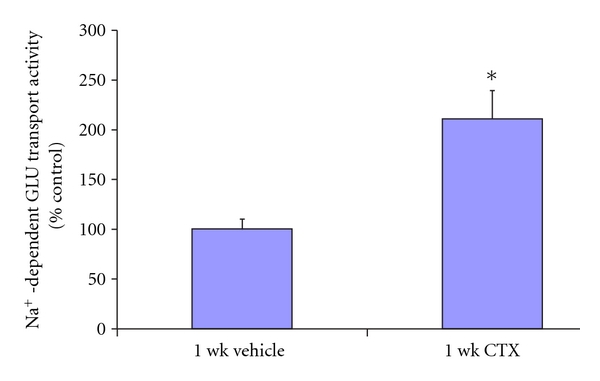
Glutamate uptake activity comparison from synaptosomes of lumbosacral spinal cord of mice administered PBS (*n* = 6) or AAV9-GLT-1 (*n* = 6) at 19 d by procedures described in Section 2. The data show a 111% increase in glutamate uptake in AAV9-GLT-1-treated animals compared to the control group. *P < 0.05.

The present study assessed the ability of transfection of AAV9-GLT-1 constructs to assess if attenuation of visceral nociception occurs. Neonatal mice were injected with 4 × 10^−11^ dps of AAV9-GLT or vehicle at 19–26 d, and the visceromotor response (VMR) to 60 mmHg colorectal distension (CRD) was measured in these animals at 8 weeks of age. The data show a 62% reduction in the nociceptive response to 60 mmHg pressure after transfection with AAV9-GLT-1 (*n* = 10) compared to animals injected with PBS (*n* = 10) (Figure 8; *P* < 0.05). This is correlated with a 111% enhanced glutamate uptake seen in AAV9-GLT-injected animals compared to controls (Figure 9).

## 4. Discussion

The principal findings of this study were (1) a spinal site of action of GLT-1 overexpression to attenuate visceral nociception, (2) effectiveness of GLT-1 overexpression to attenuate inflammogen-enhanced visceral nociception via both a preemptive and therapeutic approach, (3) CTX-mediated GLT-1 overexpression but not its anti-inflammatory effects mediates its antinociceptive effects, (4) translationally superior adenoassociated virus 9-mediated GLT-1 transfection attenuates visceral nociception to 60 mmHg colorectal distension, and (5) alterations in locomotor function were not produced by 1-week CTX treatment.

Clear evidence by our laboratory and others demonstrates the effectiveness of 1-week CTX to enhance spinal GLT-1 expression and glutamate uptake activity [4, 6, 12]. Intrathecal delivery of the selective GLT-1 antagonist DHK reversed the CTX-blunted visceromotor response to colonic distension (Figure 1). This result supports the hypothesis that upregulation of GLT-1 is responsible for the attenuated visceromotor response to colorectal distension. These findings are supported by previous work which shows that (1) intrathecal delivery of CTX produced antinociceptive effect in both thermal and mechanical nociception tests in a neuropathic pain model [4], (2) gene transfer of GLT-1 into spinal cord astrocytes by recombinant adenoviruses attenuated inflammatory and neuropathic pain, probably via prevention of central sensitization [5], and (3) intrathecal injection of GLT-1 antisense oligodeoxynucleotides reversed the antinociceptive effects of 1-week CTX treatment in a neuropathic pain model [4]. 

Intracisternal injection of doses of DHK effective after intrathecal administration (3.0 and 0.3 mM) was ineffective to reverse the reduced visceromotor response to colorectal distension produced by 1-week CTX treatment. This suggests that antagonizing brainstem glutamate transporters does not alter CTX-blunted visceral nociception. Although the volume of the fourth ventricle is larger than the intrathecal space, the concentrations of DHK given intracisternally was 1-2 orders of magnitude greater than the IC 50 of DHK (0.045 mM). These experiments support the exclusion of a role of brainstem glutamate transporters in mediating the antinociceptive effects of CTX. However, a role of midbrain, forebrain, or cerebral glutamate transporters in mediating antinociceptive effects of 1-week systemic CTX cannot be ruled out.

These experiments show that GLT-1 upregulation by one-week CTX treatment effectively reduced visceral hypersensitivity in two animal models of colitis. The myeloperoxidase (MPO) assay is a well-accepted tool to measure tissue inflammatory damage in animal models of colitis. MPO is a peroxidase enzyme abundant in neutrophils and is released upon neutrophil activation by inflammatory pathogens [19]. During inflammation, the synthesis and release of MPO are greatly enhanced. MPO has a unique ability to catalyze chloride and H_2_O_2_ to form hypochlorous acid, a powerful antimicrobial reagent. This unique property is widely used to detect the amount of MPO in tissue samples by measuring absorbance at 460 nm. 

To study potential effectiveness of the intervention of GLT-1 upregulation during an active inflammatory process, the use of DSS was employed due to the published unreliability of TNBS to produce consistent long-term inflammation in murine models [24]. In both the TNBS- and DSS- induced colitis models, GLT-1 upregulation by one-week CTX treatment was sufficient to mitigate the visceromotor response to colorectal distention (Figures 3 and 6). TNBS is a hapten that evokes an acquired immune response [24], whereas DSS-induced colitis depends on innate immunity [20]. With regards to the role of the anti-inflammatory effect of CTX itself to mitigate visceral nociception, two lines of evidence suggest a lack on an important role: (1) in the DSS model, GLT-1 overexpression had significant antinociceptive effects without significantly attenuating inflammatory effects of DSS (Figure 6 and Table 3) and (2) 1-week cephalothin, administered in equimolar doses as CTX,c produced significant anti-inflammatory effects in TNBS-treated animals (Table 2), but no reduction in the visceromotor response to colorectal distension (Figure 5). 

Probiotics are emerging as potential treatments of visceral hypersensitivity [25]. Recent studies suggest that increase in lactobacilli or its therapeutic administration produces antinociceptive effects [21]. The fact that CTX reduces lactobacilli while also producing antinociception suggests a lack of association between the two events.

Potential clinical utility of the approach to augment glutamate transporter activity depends on the lack of potential untoward effects. The rotarod test demonstrated that GLT-1 upregulation by one-week CTX treatment did not alter motor function, which is consistent with previous studies showing that CTX treatment did not change performance in the plus-maze test and open-field test, in normal mice [7].

The adenoassociated viral vector (sc AAV9) utilized in our proposal produces long-term gene expression, thus is effective after one-time injection [23]. This factor is also non-immunogenic, and it has shown promise in large animal species, including monkeys, and as recently reported [11] no toxicity was noted in treated animals. The work by Maeda et al. [5] provided assurance that gene transfer of GLT-1 will work in relieving pain, but the adenovirus used in that paper activates the immune system and produces only short-term gene transfer, thus requiring multiple injections, and thus has limited clinically relevance. Indeed, in some clinical trials, these agents have produced mortality [23]. The work performed here regarding GLT-1 transfection represents an advance in the use of a significantly superior viral vector technology, adenoassociated viral vector (sc AAV9). Selective targeting of transfection to spinal sites of action would improve translational prospectus of this approach.

Thus, the data taken together suggests that the antinociceptive effect of one-week CTX treatment is independent of its anti-inflammatory or probiotic effects and indicates the importance of GLT-1 upregulation in reducing visceral nociception. Beneficial effects are likely related to reduced extracellular glutamate tone; thus, less activation of glutamate receptors mediating nociception, and/or second messenger events downstream are attenuated (MAP kinase activity, AMPA receptor trafficking, etc.) [22, 26, 27]. 

These results support consideration of novel approach of glutamate transporter upregulation in pain therapeutics. Further investigation of ceftriaxone and analogues with increased and more specific therapeutic effects seem warranted.

## Figures and Tables

**Table 1:** Colonic myeloperoxidase (MPO) tissue levels 1 week after intracolonic (i-colo) TNBS; effect of concomitant ceftriaxone (CTX).

Treatment (*n*)	Mean ± SEM (units/mg)
1 wk veh + i-colo veh [22]	0.23 ± 0.05
1 wk CTX + i-colo veh [22]	0.18 ± 0.03
1 wk veh + i-colo TNBS [13]	0.51 ± 0.04^a^
1 wk CTX + i-colo TNBS [13]	0.23 ± 0.04^b^

One-week CTX treatment (200 mg/Kg/day (302 *μ*mole), ip) attenuated colonic inflammation caused by intracolonic (i-colo) TNBS instillation, as measured by MPO activity. ^a^ *P* < 0.005 compared to 1-week veh + i-colo veh; ^b^ *P* < 0.005 compared to 1-week veh + i-colo TNBS).

**Table 2:** Colonic myeloperoxidase (MPO) issue levels 1 week after intracolonic TNBS; effect of concomitant cephalothin (CLT).

Treatment (*n*)	Mean ± SEM (units/mg)
1 wk veh + i-colo veh [23]	0.24 ± 0.03
1 wk CLT + i-colo veh [23]	0.23 ± 0.03
1 wk veh + i-colo TNBS [23]	0.56 ± 0.09^a^
1 wk CLT + i-colo TNBS [23]	0.29 ± 0.04^b^

One-week cephalothin treatment ((302 *μ*mole) 126 mg/Kg/day, ip) was effective to attenuate colonic inflammation caused by intracolonic (ic) TNBS instillation, as measured by MPO activity. ^a^ *P* < 0.005 compared to 1-week veh + ic veh; ^b^ *P* < 0.005 compared to 1-week veh + ic TNBS).

**Table 3:** Colonic myeloperoxidase (MPO) issue levels after oral 4% DSS; effect of therapeutic ceftriaxone.

Treatment (*n*)	Mean ± SEM (units/mg)
1 wk veh + oral H_2_O [4]	0.21 ± 0.01
1 wk CTX + oral H_2_O [11]	0.17 ± 0.03
1 wk veh + 4% DSS [4]	0.50 ± 0.06^a^
1 wk CTX + 4% DSS [4]	0.35 ± 0.05^a^

Ceftriaxone (200 mg/kg daily for 1 week), administered one week after initiation of colonic inflammation by oral 4% DSS in the drinking water, was ineffective to significantly reduce colonic inflammation, measured by MPO activity at day 14. ^a^ *P* < 0.005 compared to 1-week veh + oral H_2_O.
